# Combining Pathway Identification and Breast Cancer Survival Prediction via Screening-Network Methods

**DOI:** 10.3389/fgene.2018.00206

**Published:** 2018-06-14

**Authors:** Antonella Iuliano, Annalisa Occhipinti, Claudia Angelini, Italia De Feis, Pietro Liò

**Affiliations:** ^1^Istituto per le Applicazioni del Calcolo “Mauro Picone”, Consiglio Nazionale delle Ricerche, Naples, Italy; ^2^Telethon Institute of Genetics and Medicine, Pozzuoli, Italy; ^3^Computer Laboratory, University of Cambridge, Cambridge, United Kingdom

**Keywords:** breast cancer, cox regression, high-dimensionality, network-penalized methods, screening techniques, survival analysis, pathway analysis

## Abstract

Breast cancer is one of the most common invasive tumors causing high mortality among women. It is characterized by high heterogeneity regarding its biological and clinical characteristics. Several high-throughput assays have been used to collect genome-wide information for many patients in large collaborative studies. This knowledge has improved our understanding of its biology and led to new methods of diagnosing and treating the disease. In particular, system biology has become a valid approach to obtain better insights into breast cancer biological mechanisms. A crucial component of current research lies in identifying novel biomarkers that can be predictive for breast cancer patient prognosis on the basis of the molecular signature of the tumor sample. However, the high dimension and low sample size of data greatly increase the difficulty of cancer survival analysis demanding for the development of *ad-hoc* statistical methods. In this work, we propose novel screening-network methods that predict patient survival outcome by screening key survival-related genes and we assess the capability of the proposed approaches using METABRIC dataset. In particular, we first identify a subset of genes by using variable screening techniques on gene expression data. Then, we perform Cox regression analysis by incorporating network information associated with the selected subset of genes. The novelty of this work consists in the improved prediction of survival responses due to the different types of screenings (i.e., a biomedical-driven, data-driven and a combination of the two) before building the network-penalized model. Indeed, the combination of the two screening approaches allows us to use the available biological knowledge on breast cancer and complement it with additional information emerging from the data used for the analysis. Moreover, we also illustrate how to extend the proposed approaches to integrate an additional omic layer, such as copy number aberrations, and we show that such strategies can further improve our prediction capabilities. In conclusion, our approaches allow to discriminate patients in high-and low-risk groups using few potential biomarkers and therefore, can help clinicians to provide more precise prognoses and to facilitate the subsequent clinical management of patients at risk of disease.

## 1. Introduction

Understanding the multidimensional complexity of breast cancer is an ongoing pursuit for many researchers to model survival oncological data. Technology advances offer great opportunities to explain cancer mechanisms, although there are significant challenges in extracting knowledge from such massive data and evaluating the findings. In the last years, a huge amount of genome-wide data collected using a variety of high-throughput technologies has been made publically available thanks to the effort of several international projects and consortia. For example, The Cancer Genome Atlas (TCGA) (Network, 2011, 2012, 2013), the Catalog of Somatic Mutations in Cancer (COSMIC) (Forbes et al., 2010), The Molecular Taxonomy of Breast Cancer International Consortium (METABRIC) (Curtis et al., 2012) and many others projects were established to profile large tumor sets for different omics layers, such as gene expression, DNA structure and methylation, etc. By using these types of data, biological interaction networks based on physical interactions, such as protein-protein interactions, protein-DNA interactions, and phosphorylation can be also constructed. In particular, functional interaction networks connect genes with similar or related functions and are typically inferred from multiple sources, including co-expression, KEGG pathways, functional linkage, Gene Ontology (GO) terms, etc. Overall, data from these databases not only allow to better understand the deregulation of cellular mechanisms during diseases progression, but also provide opportunities for developing novel statistical and computational algorithms for the analysis of patient omic data and for the interpretation and validation of results.

Despite this progress, many cancer diseases do not have effective treatments yet. Recently, precision medicine has been used by clinicians to take all kinds of decisions regarding the patient management and therapeutic treatments (Huang et al., 2016). In particular, prognostic biomarkers have been used for more effective selection of patient subgroups with different therapeutic strategies. In the recent past, inference was carried out by looking at a specific omic type, such as gene expression or DNA structural variations, etc. Nowadays, it is clear that multi-omic data integration is becoming necessary to investigate the genomic mechanisms involved in complex diseases (Angelini and Costa, 2014). From a statistical point of view, one of the most important challenges in integrating multi-omic profiles is to cope with the high-dimensionality of the data. To overcome this issue and optimize model predictions, innovative statistical approaches have been developed (Pineda et al., 2015). Indeed, taking more levels into account increases the dimensionality of the problem and requires additional steps for data compatibility, normalization, correction and integration (Ritchie et al., 2015; Bersanelli et al., 2016).

To reduce dimensionality from a high to a moderate scale, one can use feature screening by ranking the significant genes based on their marginal associations with the outcome variable and removing unimportant genes from the bottom of an ordered list. Feature screening techniques in ultrahigh-dimensional data analysis were introduced in Fan and Lv (2008), where the sure independence screening (SIS) and the SIS screening were proposed when the data come from an ordinary linear model with normal errors. Then, such techniques were extended to generalized linear models (Fan et al., 2009, 2010b). Nonparametric independence screening in sparse ultrahigh-dimensional additive models was presented in Fan et al. (2011). In that article, the authors suggested estimating the nonparametric components marginally with spline approximation, and ranking the importance of predictors using the magnitude of nonparametric components. A sure independent ranking and screening (SIRS) procedure to screen significant predictors in multi-index models was proposed in Zhu et al. (2011). The authors showed that under the assumption of linearity on the predictor vector, the SIRS satisfies the ranking consistency property. Finally, a sure screening procedure for Cox's proportional hazards model was presented in Fan et al. (2010a), Zhao and Li (2012) and Song et al. (2014) in order to understand the association between genomic information and survival information on oncological patients. In this work, we present three screening inspired approaches that turn out to be useful in reducing the dimensionality of the data.

Nevertheless, when the number of variables (i.e., genes or genomic features) *p* is much larger than the observations (i.e., patients) *n* (*p*≫*n*), the Cox model (Cox, 1972) cannot be applied directly. Therefore, alternative methods combining penalized Cox regression models and variable selection have been developed. Those methods include ℓ_1_ and ℓ_2_ norms (Zou and Hastie, 2005; Simon et al., 2011; Wu, 2012), the SCAD (Fan and Li, 2001), the adaptive Lasso (Zou, 2006) and the Dantzig selector (Candes and Tao, 2007) which have been proposed to infer parameters in order to further reduce the feature space and to impose sparsity on the solutions. Such penalized approaches improve prediction capabilities and interpretability of results when a large number of variables is present. Similar approaches might also be used when there exist an underlying structure on the gene/feature space, for example to account for gene regulatory mechanisms or patterns of co-expressions. In this framework, the correlation structure can be specified as constraints to the Cox model (Zhang et al., 2013; Fröhlich, 2014; Gong et al., 2014; Sun et al., 2014). Therefore, the regulatory genomic information is encoded by a network, where genes are depicted as nodes and their pair-wise relations as edges connecting two nodes. The network is converted in a Laplacian matrix and is used as penalty in the Cox regression models. In particular, the network can represent different types of relationships such as gene expression correlations, KEGG pathways information, functional interaction networks or Protein-Protein Interaction networks. Generally, the Cox regression models built on biological networks are called “network-based Cox regression models”. For instance, a comprehensive overview of computational methods used for biomarkers identification, including rank-based feature selection methods and major network methodologies used in system biology was performed in Guo and Wan (2014).

**Figure 1:**
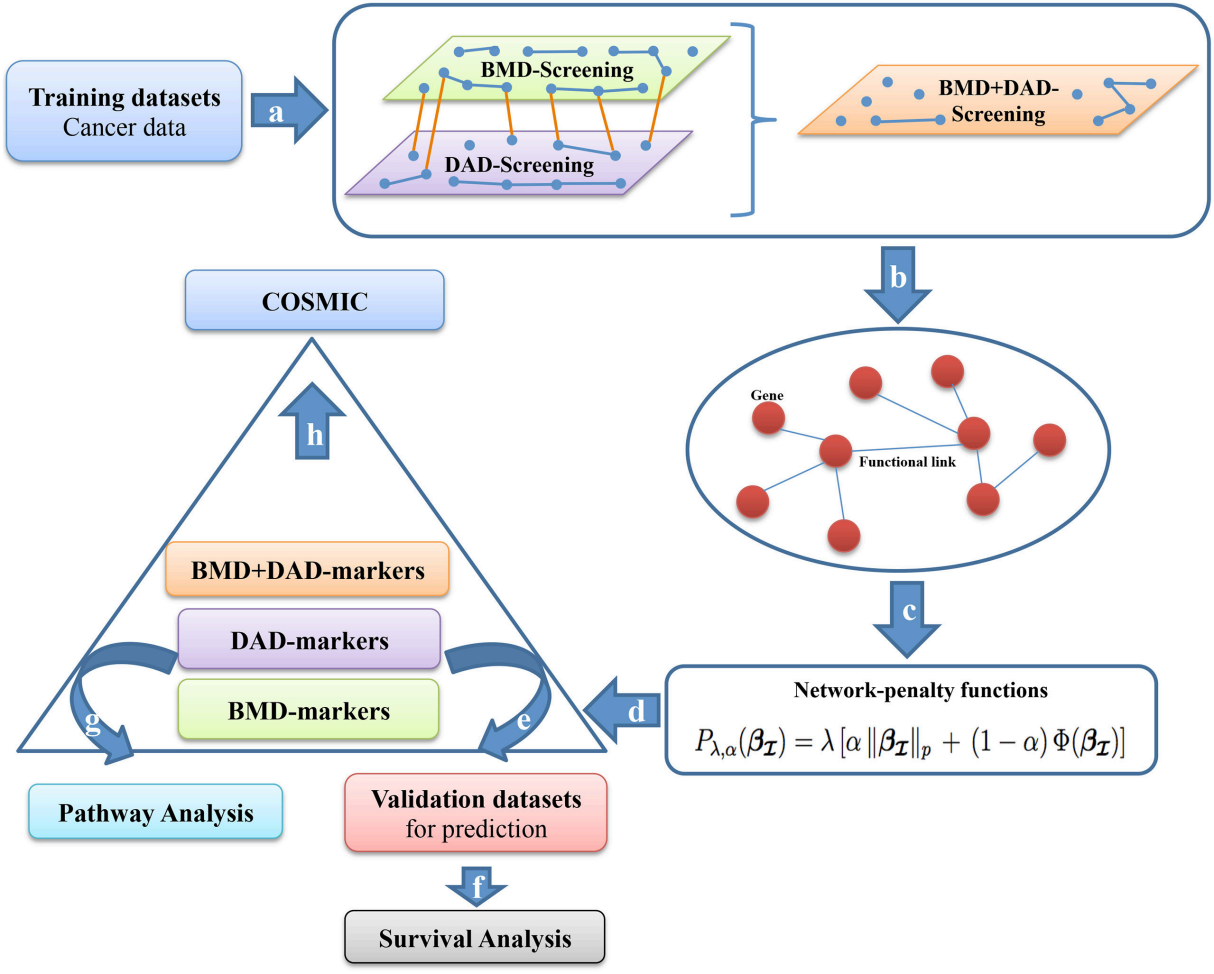
Screening-network procedure. (a) BMD-screening, DAD-screening or BMD+DAD-screening are applied to the training dataset *T* to choose a subset of genes $\left\{x_{j},j\in I\right\}$. (b) We use subset $\left\{x_{j},j\in I\right\}$ to incorporate the biological information as networks of interactions. (c) Network-penalized methods are executed on $\left\{x_{j},j\in I\right\}$. (d) High-risk genes or potential biomarkers (${\hat{\beta }}_{I}\neq 0$) are selected (from each screening approach) and are used to separate patients in two groups (high-and-low risk group). (e–f) The performance of the survival prediction are assessed using a testing dataset *D*. To make sense of the gene signatures (g) Pathway analysis is performed on high-risk genes and (h) COSMIC investigation is carried out.

In this article, we combine screening techniques and network-penalized approaches for building novel methods that select subsets of genes associated to patients survival in cancer (see Figure 1). In particular, we use METABRIC training set containing a long-term follow-up of about 1,000 breast cancer patients (Curtis et al., 2012), after having applied different types of screenings, we fit a network-penalized model for identifying gene signatures predicting survival responses. Then, we validate the capabilities of the proposed methods using about other 1,000 breast cancer patients, from METABRIC testing set. The selected gene signature provides a powerful tool for the identification of patients at high-risk of death. We also describe how to extend the proposed approaches to integrate an additional omic layer, such as copy number aberrations, and we demonstrate that such strategies can further improve our prediction capabilities. We stress that although the retrieved signatures are specific for breast cancer survival, the proposed approaches can be used for different types of cancers.

More precisely, we propose new multistage computational-statistical strategies for survival analysis based on the following steps (see Figure 1). First, we reduce the high-dimensionality of data by using one of the following types of dimension reduction techniques: (i) a biomedical-driven screening (BMD-screening); (ii) a data-driven screening (DAD-screening); (iii) a combination of BMD-and-DAD-screening (BMD+DAD-screening). These screening approaches have different advantages and drawbacks. The BMD-screening is achieved by incorporating in the model the biomedical knowledge available in literature about breast cancer and, obviously, it can be performed only when there is enough evidence available. Nowadays such information can be often retrieved for previous studies and public databases, although it is still far from being complete. On the contrary, the DAD-screening relies only on the observed data. Therefore, it is suggested when there is limited biomedical information available. To fill the gap between the two approaches, the BMD+DAD-screening is introduced to take advantage of the available biomedical knowledge and also to allow finding novel elements of investigation that can emerge from data. Hence, the BMD+DAD screening can be used when there is partial biological information available and novel information is expected to be present in the data under analysis. Such situation represents the most common case. Second, we used penalized Cox regression methods (such as AdaLnet and ADMMnet) to incorporate gene regulatory relationships and to select a subset of potential biomarkers. In carrying out this step we show that when the BMD+DAD-screening is used we detect novel disease risk genes that the simple BMD-screening ignores. Third, we validate the proposed procedure and we evaluate the predictive power of the selected gene signatures. Finally, we perform a pathway analysis based on the screened genes using Human Experimental/Functional Mapper (Huttenhower et al., 2009), KEGG pathways and COSMIC to make sense of the potential biomarkers for breast cancer survival. Moreover, to illustrate the advantages of multi omic integration, we first compare the performance of our approaches using only gene expression data, and then we integrate both gene expression and copy number aberrations. Our analysis shows that the integration provides a more comprehensive picture of breast cancer and improves the results.

Before concluding, we observe that in our previous work (Iuliano et al., 2016), we proposed a similar strategy based on the use of biological network into Cox regression penalized methods. That approach was similar in the spirit to the BMD-screening, discussed here. While confirming previous results using an independent dataset, the novelty of this article consists in the use of DAD and BMD+DAD screenings that allow us to extend and/or improve the performance of the proposed strategy, in the identification of novel potential biomarkers, and in the possibility of integrating multiple omic data types in a comprehensive analysis.

## 2. Methods

In this section, (i) we introduce the Cox proportional hazards model (Cox, 1972); (ii) we present the three different types of screening techniques used to reduce the feature space to a subset of significant variables; (iii) we discuss network-regularized methods for selecting gene signatures; (iv) we describe the proposed algorithm; (v) we illustrate the extension of the algorithm for the integration of two omic data layers; (vi) we show how to make sense of the retrieved gene signature by using pathway analysis, and finally (vii) we discuss details about the implementation of our algorithm.

### 2.1. Cox proportional hazards model

Let *n* be the number of subjects (patients), *T*_*i*_ and *C*_*i*_ for *i* = 1, …, *n* the survival time and the censoring time, respectively. Moreover, we denote the observed survival time as *t*_*i*_ = *min*{*T*_*i*_, *C*_*i*_}, the censoring indicator as δ_*i*_ = *I*(*T*_*i*_ ≤ *C*_*i*_) [where *I*(·) represents the indicator function], the regressor vector of *p*-variables for the *i*th subject (i.e. multi-omics observed profiles of the *i*th patient over *p* genes) as ${\text{x}}_{i}={\left(x_{i1},\ldots ,x_{ip}\right)}^{T}$, *i* = 1, …, *n*. We also assume that the survival time *T*_*i*_ and the censoring time *C*_*i*_ are conditionally independent given the regressors **x**_*i*_ and the censoring mechanism is noninformative. Hence, the observed data are represented by the triplets {(*t*_*i*_, δ_*i*_, **x**_*i*_), *i* = 1, …, *n*}.

Under the assumption of Cox regression (Cox, 1972) the hazard function *h*(*t*|**x**_*i*_) can be written as

$$\begin{array}{r} h\left(t|{\text{x}}_{i}\right)=h_{0}\left(t\right)\exp\left({\text{x}}_{i}^{T}\beta \right) \end{array}$$

where *h*_0_(*t*) represents the baseline hazard and $\beta ={\left(\beta_{1},\ldots ,\beta_{p}\right)}^{\prime }$ the vector of regression coefficients. In the classical settings, the regression parameters are estimated by maximizing the Cox's log-partial likelihood

(1) $$\begin{array}{r} \ell \left(\beta \right)=\overset{n}{\underset{i=1}{\sum }}\delta_{i}\left\{{\text{x}}_{i}^{T}\beta -\log\left[\underset{j\in R\left(t_{i}\right)}{\sum }\exp\left({\text{x}}_{j}^{T}\beta \right)\right]\right\}, \end{array}$$

where *R*(*t*_*i*_) denotes the risk set at time *t*_*i*_ (i.e., the set of all patients who still survived prior to time *t*_*i*_).

When the number of genes *p* is much larger than the patients *n* (*p* ≫ *n*), such approach cannot be applied since the solution become not identifiable. To cope with this issue, improving prediction performance and the interpretation of the data, several penalization approaches have been proposed. Such techniques consist in adding a ℓ_1_-penalty and/or ℓ_2_ penalty term to the log-likelihood (1) in order to reduce the solution space imposing sparsity and small coefficients for the parameters (Tibshirani, 1996; Tibshirani et al., 1997; Zou and Hastie, 2005).

### 2.2. Variable screenings for cox's proportional hazards model

The first step of our strategy is the variable screening of the data which aimed to reduce the number of variables for a large to a moderate scale. To this purpose, we assume that only a small number of these *p* variables is affecting the survival outcome. Therefore, we filter out variables that are considered not relevant for the disease under investigation. To this purpose, we consider three different types of variable screenings: biomedical screening (BMD-screening), data-driven screening (DAD-screening) and the fusion of biomedical and data-driven screening (BMD+DAD-screening). In the following sections, we define the set $\left\{x_{j},j\in I\right\}$ as the subset of the screened variables and $d=|\left\{x_{j},j\in I\right\}|$ its cardinality.

#### 2.2.1. Biomedical-driven screening

In this type of screening to identify the subset $\left\{x_{j},j\in I\right\}$ we used only the biological information that has been accumulated in the literature on the cancer disease under investigation (Iuliano et al., 2016) and it is available in some external databases. In particular, as source of biological information (i.e., genes potentially associated to breast cancer) we used Human Experimental/Functional Mapper (HEFaIMp) (Huttenhower et al., 2009). Such web-resource describes the genes functional activity and gene-gene interactions in over 200 areas of human cellular biology with information from 30,000 genome-scale experiments and summarizes information from different biological informative sources such as prediction of protein function and functional modules, cross-talk among biological processes, and association of novel genes and pathways with known genetic disorders. HEFaIMp provides a *p*-value for each gene that indicates how significant is the relation between the gene and the disease of interest. Hence, we define with $I_{BMD}$ the subset of genes selected by using HEFaIMp tool with *p*-value less or equal than 0.05 and with *d*_*BMD*_ its cardinality. We called this screening BMD-screening.

Note that, in the BMD-screening, we select the $I_{BMD}$ set using standard *p*-value (with significance threshold equal to 0.05) without controlling for multiple tests, because we use a two-stage procedure composed by a screening step followed by a variable selection method (i.e., the network approach described in 2.3). In this context, the identification of the variables associated to our pathology is performed in the variable selection step, and the screening is simply aimed to perform a pre-selection of the features. Such approach is typically done in the context of statistical screenings (see Fan and Lv, 2008). However, to further screen the variables of interest it could be also possible to control the multiplicity at the screening level, as described in Dmitrienko et al. (2009).

#### 2.2.2. Data-driven screening

In this type of screening to identify $I_{DAD}$ we reduce the feature space from a large scale dimension *p* to a relatively moderate scale *d* < *p* by using only information from the data. This type of knowledge consist of the matrix *X* that contains single omic or multi omics patient profiles. Such approach differ from the BMD-screening where the information on which gene filter out and which retain in the model was obtained from an external database.

Let $M_{*}=\left\{1\leq i\leq p:\beta_{i}^{*}\neq 0\right\}$ be the true sparse Cox model. The maximum marginal likelihood estimator (MMLE) $\beta_{k}^{M}$, for *k* = 1, …, *p*, is defined in Cox model as the maximizer of the log-partial likelihood with a single covariate

(2) $$\begin{array}{r} \beta_{k}^{M}={\text{arg max}}_{\beta_{k}}\overset{n}{\underset{i=1}{\sum }}\delta_{i}\left\{x_{ki}\beta_{k}-\log\left[\underset{j\in R\left(t_{i}\right)}{\sum }\exp\left(x_{kj}\beta_{k}\right)\right]\right\}. \end{array}$$

The component-wise estimators can be computed very rapidly and implemented modularly, avoiding the numerical instability associated with ultrahigh dimensional estimation problems. The SIS procedure ranks the importance of features according to the magnitude of their marginal regression coefficients. Therefore, we select a set of variables

(3) $$\begin{array}{r} I_{DAD}=\left\{1\leq k\leq p:|\beta_{k}^{M}|\geq \delta_{n}\right\} \end{array}$$

where δ_*n*_ is a threshold value chosen so that we pick the *d*_*DAD*_ top ranked covariates. The higher correlation, the higher the ranking position. As often suggested, one may choose ⌊*n*log*n*⌋ as threshold to select the most appropriate number of genes to retain in the model. More in general, the choice of the threshold may also be either data-driven or model-based. However, the aim of the screening procedure is to filter out as many noisy variables as possible, retaining all interesting ones in the model. After that the penalty in the network-based approach will select the few most relevant features.

For this reason, in our study we select different thresholds and we study their effect to optimize data prediction. It is easy to note that larger *d*_*DAD*_ means larger probability of including the true model $M_{*}$ in the final model with indices in $I_{DAD}$. We called this screening DAD-screening.

#### 2.2.3. Biomedical-driven and data-driven screening

In this type of screening to identify $I_{BMD+DAD}$ we merge the biological information known in literature and the data-driven knowledge to obtain new insights about cancer diseases by taking the union of the BMD and DAD sets of genes, i.e., *d*_*BMD*+*DAD*_ = *d*_*BMD*_ ∪ *d*_*DAD*_. Indeed, no cancer has been yet fully characterized in term of disrupted genes and/or metabolic processes involved in the disease. In particular, the BMD and DAD screenings take into account respectively available biological knowledge (i.e., genes highly correlated to breast cancer as described in the literature) and genes closely associated with the survival response (as emerging from the data). The BMD and DAD screening represent two faces of the same medal and naturally complement themselves. By using BMD+DAD screening, we aim to explore the best model that can sufficiently explain the data in the most parsimonious way in order to (i) make use of available information, (ii) identify new markers that the BMD-screening ignores, and (iii) improve the ability to make precise prognosis, diagnosis and treatments. In fact, although breast cancer is the most common cancer types analyzed in literature, still remains a need for a more comprehensive and exhaustive study to find and investigate novel biomarkers. We called this screening BMD+DAD-screening.

### 2.3. Network approaches after screening

The second step of our strategy is the application of penalized methods using the subset of screened variables $\left\{x_{j},j\in I\right\}$ (where I depends on the type screening performed) as new feature space to further remove not significant variables from the model. The Cox penalized partial likelihood is

(4) $$\begin{array}{l} \ell (\beta_{I})=\text{arg}{\text{min}}_{\beta_{I}}(\sum_{i=1}^{n}\delta_{i}\{x_{I,i}^{T}\beta_{I}\\ \;-\log\left[\sum_{j\in R(t_{i})}\exp\left(x_{I,j}^{T}\beta_{I}\right)\right]\}+P_{\lambda }\left(\beta_{I}\right)), \end{array}$$

where ${\text{x}}_{I,i}^{T}$ denotes a sub-vector of **x**_*i*_ with indices in I, see Equation (3). $\beta_{I}$ are the screened regression coefficients. In particular, we add a penalty function $P_{\lambda }\left(\beta_{I}\right)$ on the regression coefficients $\beta_{I}$. In the following section we introduce network-penalized approaches on the screened genes $\left\{x_{j},j\in I\right\}$ to incorporate an a-priori biological knowledge into the model and to predict survival outcomes.

#### 2.3.1. Network-regularized cox regression

The existing relationships among the covariates can be described in terms of a weighted and undirected graph (network) *G* = (*V, E, W*) where the vertices *V* = {1, …, *d*} represents genes or covariates, an element (*i, j*) in the edge set *E* ⊂ *V* × *V* indicates a relationship between vertices *i* and *j*. *W* = (*w*_*ij*_), (*i, j*)∈*E* represent the weights (or strength of the relationship) associated with the corresponding edges. The relationships between genes can be obtained in terms of gene-gene interaction, KEGG pathway analysis or protein-protein interaction, or other functional information and it is normalized in [0, 1] where 0 indicates an absence of relationship and 1 a strong relationship. More in general, the weight may indicate the probability that two genes are functionally connected. Such information is incorporated in the analysis using a penalty function $P_{\lambda }\left(\beta_{I}\right)$ in Equation (4).

More formally, we introduce the following network penalty function

(5) $$\begin{array}{r} P_{\lambda ,\alpha }\left(\beta_{I}\right)=\lambda \left[\alpha ∥\beta_{I}{∥}_{p}+\left(1-\alpha \right)\Phi \left(\beta_{I}\right)\right] \end{array}$$

where λ > 0 (sparsity) and α ∈ (0, 1] (network influence) are two regularization parameters (Zhang et al., 2013; Guo and Wan, 2014). The subset I includes the variables selected by using the previous screening approaches (BMD-screening, DAD-screening or BMD+DAD-screening). The penalty function is composed by two terms. The first part is a ℓ_*p*_-norm with *p*∈{1, 2} which induces sparsity or thresholding; the second one Φ(·) is a Laplacian matrix constraint which gives smoothness among two adjacent coefficients in the network. Generally, Φ(·) for every pair of genes linked by an edge, which is proportional to the edge weight and the difference between their coefficients is a cost function. This hypothesis indicates that the two genes should be correlated. In other words, the regression coefficients should be similar, i.e., vary smoothly through the network (Zhang et al., 2013; Sun et al., 2014).

In our work, we use two of the most recent network-based Cox regression models. The details of each method are listed below.

The first method is based on a-priori network information is *Ada*ptive *L*aplacian *net* (or *AdaLnet*) (Sun et al., 2014). Denoting with $d_{i}=\underset{i:\left(i,j\right)\in E}{\sum }w_{ij}$ the degree of vertex *i*, *AdaLnet* defines the normalized Laplacian matrix **L** = (*l*_*ij*_) of the graph *G* (positive semi-definite) by

$$\begin{array}{c} l_{i,j}=\{\begin{array}{ll} 1, & \text{if }i=j\;\text{and }d_{i}\neq 0,\\ -\frac{w_{ij}}{\sqrt{d_{i}d_{j}}}, & \text{if}(i,j)\in E,\\ 0, & \text{otherwise}. \end{array} \end{array}$$

The network-constrained penalty in Equation (4) is given by

(6) $$\begin{array}{r} P_{\lambda ,\alpha }\left(\beta_{I}\right)=\lambda \left[\alpha ∥\beta_{I}{∥}_{1}+\left(1-\alpha \right)\Phi \left(\beta_{I}\right)\right], \end{array}$$

where

$$\begin{array}{r} \Phi \left(\beta_{I}\right)=\underset{\left(i,j\right)\in E}{\sum }w_{i,j}{\left(\frac{sgn\left({\tilde{\beta }}_{i,I}\right)\beta_{i,I}}{\sqrt{d_{i}}}-\frac{sgn\left({\tilde{\beta }}_{j,I}\right)\beta_{j,I}}{\sqrt{d_{j}}}\right)}^{2}. \end{array}$$

The penalty in Equation (6) is the sum of an ℓ_1_-penalty that brings sparsity and a quadratic Laplacian penalty that induces smoothness between adjacent vertices in the network. The vector ${\tilde{\beta }}_{I}$ is obtained from a preliminary regression analysis. The scaling of the coefficients $\beta_{I}$ respect to the degree allows the genes with more connections (i.e., the hub genes) to have larger coefficients. Hence, small changes of expression levels of these genes can lead to large changes in the response. An advantage of using the penalty in Equation (6) consists in representing the case when two neighboring variables have opposite regression coefficient signs, which is reasonable in network-based analysis of gene expression data. Indeed, when a transcription factor (TF) positively regulate gene *i* and negatively regulate gene *j* in a certain pathway, the corresponding coefficients will result with opposite sign.

Note that, here λ is the parameter that regularizes by the likelihood network constraint and α∈(0, 1] is the parameter balancing the network constraint with respect to the sparsity.

The second network penalized method is based on the Alternating Direction Method of Multipliers (ADMM) algorithm used to solve a broad range of statistical optimization problems (Boyd et al., 2011). ADMM is an algorithm that solves convex optimization problems by breaking them into smaller pieces, each of which are then easier to handle. The algorithm solves problems in the form:

(7) $$\begin{array}{r} \text{minimize }f\left(\text{x}\right)+g\left(\text{z}\right)\text{ subjectto }\text{A}\text{x}+\text{B}\text{z}=\text{c} \end{array}$$

with **x** ∈ ℝ^*n*×1^, **z** ∈ ℝ^*m*×1^, **A** ∈ ℝ^*p*×*n*^, **B** ∈ ℝ^*p*×*m*^, and **c** ∈ ℝ^*p*×1^. The functions *f* and *g* are supposed convex. The optimal value of the problem Equation (7) will be denoted by

$$\begin{array}{l} p^{*}=\text{inf}\left\{f\left(\text{x}\right)+g\left(\text{z}\right)|\text{A}\text{x}+\text{B}\text{z}=\text{c}\right\}. \end{array}$$

An alternative formulation is the following Lagrangian form

(8) $$\begin{array}{r} L_{\rho }\left(\text{x},\text{y},\text{z}\right)=f\left(\text{x}\right)+g\left(\text{z}\right)+{\text{y}}^{T}\left(\text{A}\text{x}+\text{B}\text{z}-\text{c}\right)+\left(\rho /2\right)∥\text{A}\text{x}+\text{B}\text{z}-\text{c}{∥}_{2}^{2} \end{array}$$

ADMM consists of the iterations:

(9) $$\begin{array}{r} \begin{array}{c} {\text{x}}^{k+1}:=\text{argmin}L_{\rho }\left(\text{x},{\text{z}}^{k},{\text{y}}^{k}\right),\\ {\text{z}}^{k+1}:=\text{argmin}L_{\rho }\left({\text{x}}^{k+1},\text{z},{\text{y}}^{k}\right),\\ {\text{y}}^{k+1}:={\text{y}}^{k}+\rho \left(\text{A}{\text{x}}^{k+1}+{\text{B}\text{z}}^{k+1}-\text{c}\right). \end{array} \end{array}$$

where ρ>0. The algorithm consists of an *x*-minimization step, a *z*-minimization step, and a dual variable update (see Equation 9). Therefore, the method of multipliers for solving the problem in Equation (9) has the form

$$\left({\text{x}}^{k+1},{\text{z}}^{k+1}\right):={\text{argmin}}_{\text{x},\text{z}}L_{\rho }\left(\text{x},\text{z},{\text{y}}^{k}\right)$$

$${\text{y}}^{k+1}:={\text{y}}^{k}+\rho \left({\text{A}\text{x}}^{k+1}+{\text{B}\text{z}}^{k+1}-\text{c}\right).$$

The algorithm state in ADMM consists of **z**^*k*^ and **y**^*k*^, i.e. (**z**^*k*+1^, **y**^*k*+1^) is a function of (**z**^*k*^, **y**^*k*^). In ADMM form, our problem can be written as

(10) $$\begin{array}{r} \text{minimize }f\left(\text{x}\right)+g\left(\text{z}\right)\text{ subjectto }\text{x}-\text{z}=0 \end{array}$$

where $f\left(\text{x}\right)=\ell \left(\beta_{I}\right)$ (Equation 1) and $g\left(\text{z}\right)=P_{\lambda ,\alpha }\left(\beta_{I}\right)$ (Equation 5 with *p* = 1) evaluated on $\beta_{I}$. The network penalty function is given by

$$P_{\lambda ,\alpha }\left(\beta_{I}\right)=\lambda \left[\alpha ∥\beta_{I}{∥}_{1}+\left(1-\alpha \right)\phi \left(\beta_{I}\right)\right],$$

where $\phi \left(\beta_{I}\right)=\beta^{T}L\beta$ with *L* is the Laplacian matrix.

#### 2.3.2. Laplacian matrix

The Laplacian matrix that describes the functional relationships among genes is constructed as done in our previous work (Iuliano et al., 2016) for genes covered by HEFalMp tool; the matrix is completed by adding zero weights for all the genes that are non-covered by HEFaIMp.

#### 2.3.3. Tuning parameters by k-fold cross-validation

In principle, cross-validation can be used for estimating both α and λ. However the global procedure can be time-consuming and often provide only a limited improvement with respect to the optimization carried out using only one parameter. Therefore, we fixed α = 0.5 and we estimate λ using cross-validation. To better understand the rationale of such choice, we note that α∈[0, 1] represents the influence of the network in the model. Small values of α will result in no network influence, large values α indicate strong influence. The choice α = 0.5 assumes moderate influence of the network and represents a standard default parameter.

In order to estimate λ, the dataset is partitioned in *K* = 5 different folds, where four parts are used for finding model's coefficients ${\hat{\beta }}_{\left(\lambda ,\alpha \right)}^{\left(-k\right)}$ and one part is used for assessing the prediction on unseen data. This procedure is repeated 5 times, shuffling the folds. The estimate is obtained by maximizing the cross-validation log-partial likelihood (CVPL) defined as

$$\begin{array}{r} CVPL\left(\lambda ,\alpha \right)=-\frac{1}{n}\overset{K}{\underset{k=1}{\sum }}\left\{\ell \left({\hat{\beta }}_{\left(\lambda ,\alpha \right)}^{\left(-k\right)}\right)-\ell^{\left(-k\right)}\left({\hat{\beta }}_{\left(\lambda ,\alpha \right)}^{\left(-k\right)}\right)\right\}, \end{array}$$

where ${\hat{\beta }}^{\left(-k\right)}\left(\cdot \right)$ is the estimate obtained from excluding the *k*th part of the data with a given pair of (λ, α), ℓ(·) is the Cox log-partial likelihood on all the sample and ℓ^(−*k*)^(·) is the log-partial likelihood when the *k*th fold is left out (van Houwelingen et al., 2006). To assess the stability of the survival prediction we performed the five-fold cross-validation 10 times and we take as estimate the average value of λ.

#### 2.3.4. Survival analysis

The results of section 2.3.1 consist in a gene signature, i.e., ${\hat{\beta }}_{I}\neq 0$, that can be used to predict patient survival. Survival analysis is performed using the Kaplan Meier curves after dividing the patients in two risk groups (high-and-low risk group) on the basis of the prognostic index computed with the gene signature. The *p*-value, used to test the null hypothesis that the survival curves are identical vs. the alternative that the two groups have different survival, is calculated by using the log-rank test.

### 2.4. General algorithm for the screening-network survival prediction

In this section, we present the general procedure used for model's prediction.

**Algorithm 1**. *Screening-network survival prediction*.
  Let define *T* the training set and *D* the validation set.

1. Apply screening techniques on *T* to reduce the dimension of the variable space from a large scale *p* to a moderate scale *d*, *d* < *p*. BMD- or DAD- or BMD+DAD-screening can be used for such purpose.
  - Define the subset $\left\{x_{j},j\in I\right\}$ as the subset of the screened variables.
2. Perform network-based Cox regression methods on $\left\{x_{j},j\in I\right\}$ in order to select the high-risk cancer genes. Either AdaLnet or ADMM can be used in this step.
  - Fix the regularization parameter α = 0.5 to assess the network influence.
  - Repeat five-fold cross validation 10 times and take the mean of this estimate as the optimal tuning parameter values $\left({\hat{\lambda }}_{I},{\hat{\alpha }}_{I}\right)$.
  - Use ${\hat{\lambda }}_{I}$ and ${\hat{\alpha }}_{I}$ to fit the corresponding penalized model and denote the parameter estimate by ${\hat{\beta }}_{I}$.
  - Select the BMD- or DAD- or BMD+DAD-genes with regression coefficients ${\hat{\beta }}_{I}\neq 0$.
3. Compute the prognostic index (PI) for each patient *i* in *T*, for *i* = 1, …, *n*, as
  (11) $$\begin{array}{r} PI_{i}^{I}={\text{x}}_{i}^{I}{\hat{\beta }}_{I}, \end{array}$$
  where ${\text{x}}_{i}^{I}$ is the vector of screened gene expression value (or adjusted expression) associated to the *i*-th patient.
  - $PI_{i}^{I}$ is used to partition the patients in two subgroups, that correspond to the high-risk and low-risk prognosis groups, as follows:
    - Compute the quantile *q*_γ_ of $PI_{i}^{I}$, with γ = 0.20, 0.25, 0.30…, 0.80.
    - Each patient *i* in *T* is assigned to the high-risk (or low-risk) group if its prognostic index $PI_{i}^{I}$ is above (or below) the *q*_γ_-quantile.
    - The optimal cutoff PI^*,*T*^ is selected adaptively on *T*. Here, the optimal cutoff is the γ-value that corresponds to the best separation in high-and-low risk group with respect to the log-rank test as defined in Iuliano et al. (2016).
4. Calculate the prognostic index $PI_{i}^{D}$ by using ${\hat{\beta }}_{I}$ and PI^*,*T*^.
  - Each patient *i* in *D* is assigned into the high/low-risk group if its prognostic index $PI_{i}^{D}={\text{x}}_{i}^{D}{\hat{\beta }}_{I}$ is above (or below) the fixed threshold PI^*,*T*^. The value ${\text{x}}_{i}^{D}$ is the vector of gene expression value associated to the *i*-th patient in *D*.
5. Perform the log-rank test to compare the survival curves between the patients in the high-risk and low-risk groups defined by the predicted risk scores $PI_{i}^{D}$.
  - The performance measure is the *p*-value of the test (the significance level was set at 5%, i.e., *p*-value < 0.05).

### 2.5. Multiomics data integration

In the above description the matrix **X** is usually a classical gene expression matrix. In order to integrate the information of an additional omic layer we use MANCIE (matrix analysis and normalization by concordant information enhancement) (Zang et al., 2016). MANCIE can be applied using two (column-matched) data matrices and adjusts one (main matrix) using the other (associated matrix) by identifying and reinforce the concordant information in the two matrices and reducing the discordant information between them. The two data matrices must contain two omic-profiles on the same set of samples/patients. For example, one can measure the same omic profile using different experimental platforms or one can consider different omic types. The main matrix refers to the type of data that is considered more relevant whose values are returned “adjusted.” In this study, MANCIE was used to adjust mRNA data matrix (main matrix) using copy number aberrations (CNAs), as the associated matrix. The resulting adjusted matrix was used in our algorithm in the case of two omics analysis.

### 2.6. Pathway analysis

Using ${\hat{\beta }}_{I}\neq 0$, we perform a pathway analysis based on KEGG database to make sense of the proposed signatures (http://www.kegg.jp/ or http://www.genome.jp/kegg/). Therefore, we associated to each gene the list of KEGG pathway in which it is annotated and the number of publications that relates it to breast cancer. We represents our results in terms of a network. In order to draw such networks we considered only the *not isolated* genes, where a gene *g* is said *not isolated* if *G* ∩ *K* ⊋ {*g*} (*G* denoting a given set of genes and *K* a given KEGG pathway). Namely, *g* is *not isolated* if there is at least another gene *g*′ ∈ *G* belonging to the same pathways of *g*. In such cases *g* and *g*′ will be connected by an edge that depend on the pathway *K*.

In this representation, each node in the network represents a gene and an edge between two nodes means that the corresponding genes belongs to the same KEGG pathway. In particular, we use different colors for different pathways and three colors to identify the type of screened gene: orange color for genes selected by HEFaIMp tool with *p*-value < 0.05, green color for genes selected by HEFaIMp tool with *p*-value >0.05, purple color for genes that are not explored by HEFaIMp tool. Triangular-shaped nodes correspond to the genes that have already been identified in literature as breast-cancer associated genes. The latter step has been done using the database available in Cotterill (1999). The number of papers that associates such genes to breast cancer is also reported in the triangular nodes.

We also use the Catalog Of Somatic Mutations In Cancer (COSMIC, v84) (Forbes et al., 2010) for exploring the impact of somatic mutations in breast cancer. We downloaded COSMIC database from https://cancer.sanger.ac.uk/cosmic/download. We analyzed genes obtained by the DAD-screening and BMD+DAD-screening.

### 2.7. Implementation of the algorithm

The statistical approach presented in Figure 1 and described in Algorithm 1 has been implemented as a comprehensive R script that allows to execute all methods under the same R environment. The METABRIC gene expression profiles (Molecular Taxonomy of Breast Cancer International Consortium) were downloaded from the European Genome-phenome Archive (EGA). Access to datasets was approved by the specified Data Access Committee (DAC). The Illumina probes were annotated with the mappings from the Bioconductor package illuminaHumanv4.db (Dunning et al., 2015). Whereas, the METABRIC copy number aberrations CNAs data were downloaded by cBioPortal for Cancer Genomics (www.cbioportal.org).

For the BMD-screening, we select a subset of genes that are involved in breast cancer by using a functional map that summarize the most relevant interactions in the cancer area of interest (Huttenhower et al., 2009). This map is used to build the network-matrix and to identify the weight of the edges among genes.

We use AdaLnet method which is a pathwise algorithm for the Cox proportional hazards model, regularized by network penalty [combination of ℓ_1_-penalty, $∥\beta_{I}{∥}_{1}$ and Laplacian matrix $\Phi \left(\beta_{I}\right)$] (Simon et al., 2011; Sun et al., 2014). It is implemented in Coxnet package (version 0.2, 2015-03-21). ADMM is an algorithm implemented in the ADMMnet package (version 0.1, 2015-12-12). For each method we fix the regularization parameter α = 0.5 and repeat five-fold cross validation 10 times. Then we take the mean of this estimate as the optimal tuning parameter values (see Algorithm 1). Then, Survival package in the R software is used to compare the Kaplan-Meier survival curves and to derive the significance *p*-value indicating the difference between two survival curves. For the integration of different omic profiles MANCIE package was used (version 1.4, 2016-03-02).

Pathways analysis has been carried out by using the KEGG database through an integrative R script. RCytoscape (www.bioconductor.org/packages/release/bioc/html/RCytoscape.html) has been used to draw the networks (Shannon et al., 2013). Note that all the scripts are available upon request from the first two authors.

## 3. Results and discussion

In this section, we present the results obtained using the proposed approaches using the METABRIC dataset. For such purpose, we divided the dataset in two parts, training set (*T*) and testing set (*D*) as described in section 3.1 We compared the three screening procedures (BMD, DAD and DAD+BMD) combined with the two network Cox regression methods (AdaLnet and ADMM) with respect to the subset of screened genes (i.e., $\left\{x_{j},j\in I\right\}$) and their cardinality *d*, the potential biomarkers identified (i.e., those with regression coefficients ${\hat{\beta }}_{I}\neq 0$), and the survival prediction capabilities. The screened genes and the potential biomarkers were evaluated on the training set, the latter resulting in a gene signature able to subdivide patients in high and low risk groups. The prediction capabilities were evaluated by using Kaplan-Maier curves and log-rank tests on the testing set. After that the list of potential biomarkers underwent to a pathway analysis in order to provide a biological interpretation of the results and illustrate the relationship with already available biological information. In discussing the results, we first show those obtained by analyzing only mRNA expression data, then we show the improvement observed by integrating mRNAs and CNAs using MANCIE as described in section 2.5. Overall our results show that the BMD+DAD-screening is better than BMD or DAD in terms of predictive power (i.e., smaller *p*-value for the log-rank test on the testing set) for breast cancer survival patients and also allows to identify as potential biomarkers few genes that the BMD screening ignores. Moreover, we also demonstrate that integrating two omic data types improves the predictions.

In the following, AdaLnet method is referred as Coxnet and ADMM is referred as ADMMnet, according to the R packages where they are implemented.

### 3.1. Data availability

We used METABRIC data to evaluate the performance of our screening-network approach. This dataset contains clinical traits, mRNA expression data, CNAs profiles, and SNP genotypes derived from 1980 breast cancer samples (patients) (Curtis et al., 2012). In particular in our comparison, we use mRNA expression data downloaded from The European Genome phenome Archive (EGA) with number EGAS00000000083 and the copy number aberrations (CNAs) available on cBioPortal for Cancer Genomics (http://www.cbioportal.org/). The mRNAs data consist in a matrix containing 48,803 Illumina expression probes measured on the Illumina HT-12 v3 platform. The CNAs matrix is coded using value −2 to indicate homozygous deletion; value −1 to represent the hemizygous deletion; value 0 meaning neutral/no change; value 1 showing the gain; value 2 for high level of amplification. Both the matrices are normalized as discussed in Curtis et al. (2012). By using these data, we conducted two types of analysis based on (i) mRNA expression data and (ii) integration mRNA and CNAs.

As a first step, we divided the patients in two subsets: a training set *T* (997 samples) and testing set *D* (995 samples). When performing the analysis using only the mRNA expression data a total of 19,151 genes was retrieved from 48,803 Illumina expression probes by using a bioconductor annotation data package (Dunning et al., 2015). When performing the analysis integrating mRNA and CNAs information a total of 18,006 genes (containing both mRNA and CNAs information) was considered from 26,298 copy number features summarized at the gene level. A summary of METABRIC dataset is shown in Table 1.

**Table 1:** METABRIC dataset summary: mRNA expression dataset and the integration of mRNA data CNAs profiles (mRNA+CNAs).

**Omics data**	**Training set (T)**		**Testing set (D)**	
	**Sample**	**# Genes**	**Sample**	**# Genes**
mRNA	997	19,151	995	19,151
mRNA+CNAs	997	18,006	995	18,006

*For each case, the samples are divided into two subsets: training set T e testing set D, respectively*.

Finally, the overall survival (OS) data related to the 1980 patients (long-term follow-up data) were downloaded from cBioPortal for Cancer Genomics (*Q*_1_ = 60.78 months, Median = *Q*_2_= 116.10 months, *Q*_3_ = 184.90 months). In particular, the OS-status indicator was divided in *died of disease* (deceased=1), *living* (censored=0) and *died of other causes* (censored=0), respectively (Gao et al., 2013).

### 3.2. Screening-network analysis

**Figure 2:**
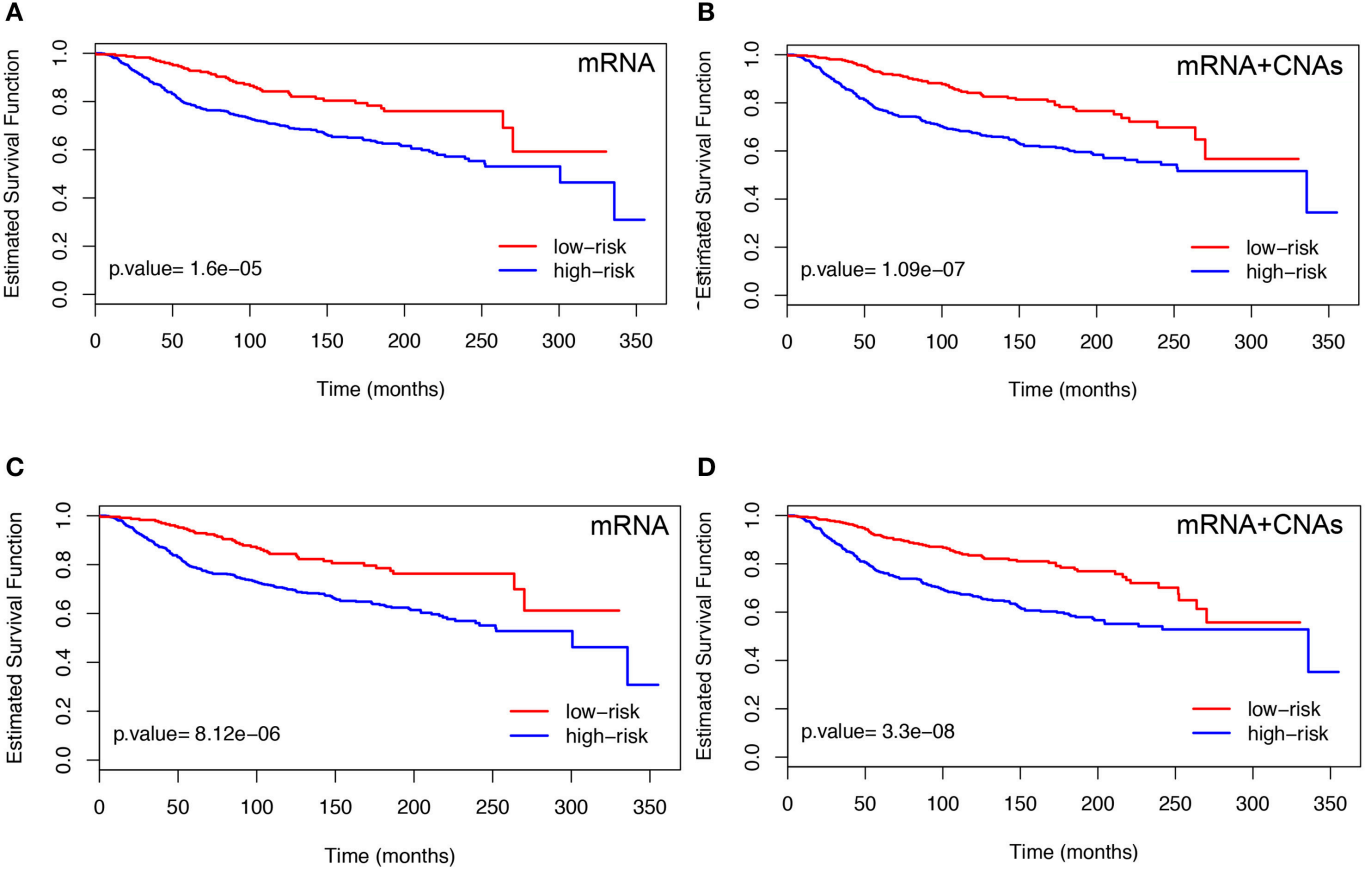
Kaplan-Meier plots obtained using mRNA data (left) and the integration of mRNA and CNAs profiles (right). The results refer to the testing set *D*. For each case, patients were divided into two groups according to the prognostic index by using BMD-genes with Coxnet **(A,B)** and ADMMnet **(C,D)**. We use the color blue to indicate the high-risk group and the color red to show the low-risk group. The *p*-value is also calculated applying the log-rank test on testing set. The high-risk group is better separated from the low-risk group by using the integration of mRNA expression data and CNAs profiles (right), compared with using the single omics data (left). The X-axis represents time and the Y-axis represents survival rate.

First, we describes the results obtained using the BMD-screening. In order to select $\left\{x_{j},j\in I_{BMD}\right\}$ we used HEFalMp tool (http://hefalmp.princeton.edu/hefalmp) and we selected only those genes that in the HEFalMp tool have *p*-value < 0.05 for breast cancer association. In particular, the BMD-screening selected a total of *d*_*BMD*_ = 528 genes when using mRNA expression data and *d*_*BMD*_ = 526 genes when integrating mRNA and CNAs data (see, Table S1 for the screened gene lists). Such subsets of genes reflect the bio-medical knowledge about breast cancer markers available from previous studies. HEFalMp was also used to build the gene network to be used in the network-penalized Cox regression method. Then, the network-based Cox regression methods applied on the training dataset, *T*, allowed us to select high-risk genes or potential biomarkers (i.e., those with regression coefficients ${\hat{\beta }}_{I_{BMD}}\neq 0$). We denoted this gene signature as BMD-genes (see Table 2 and Table S2). BMD-genes were used to compute the prognostic index of each patient and to classify them in low and high risk groups. An optimal cut-off for the prognostic index was estimated for such purpose. The significance of the BMD-gene lists was evaluated on the testing dataset, *D*, in terms of *p*-values of the log-rank test were novel patients were divided in low and high risk groups according to their prognostic index. Figure 2 shows the Kaplan-Meier survival curves on the testing set *D* for each combination between the BMD-screening and the network-penalized Cox regression methods, by using only mRNA expression data and the integration between mRNA and CNA profiles, respectively. Figures 2A,B refer to Coxnet and Figures 2C,D to ADMMnet. Table 2 shows additional results of our procedure in terms of identified markers in the training set *T* and log-rank test *p*-value obtained from the testing set *D*. Overall such results confirm those obtained in Iuliano et al. (2016) on an independent datasets. Moreover, they also show that the integration (mRNA+CNA data) of two omic types provides a better prediction of patient survival (i.e., better separation in terms of *p*-value) than the use of a single omic layer (mRNA expression data), thus extending the results of previous work.

**Table 2:** Number of BMD-genes selected by using the combination of BMD-screening and network-penalized Cox methods (Coxnet and ADMMnet) with regression coefficients ${\hat{\beta }}_{I_{BMD}}\neq 0$ on the training set *T*.

**Omics data**	**Methods**	**# BMD-genes**	**p-value**	**α**	**λ**
mRNA	Coxnet	38	1.6e-05	0.5	0.07934
	ADMMnet	43	8.12e-06	0.5	0.07695
mRNA+CNAs	Coxnet	24	1.09e-07	0.5	0.09338
	ADMMnet	19	3.3e-08	0.5	0.10170

*The tuning parameters ($\lambda_{I_{BMD}},\alpha_{I_{BMD}}$) and the relative p-values obtained from the testing set D are also shown*.

To better understand the BMD-genes signature obtained using mRNA and/or mRNA+CNAs data, we show the heatmap of the gene expression. In particular, we ordered the patients with respect to the prognostic index PI and divide them in two risk classes (i.e., low-risk and high risk) using the optimal cut-off PI^*^, as described in section 2.4. Figure S1 shows the Z-score matrix of the BMD-genes expression in the training (*T*) and testing (*D*) sets, respectively and Figure S2 shows similar heatmaps for the Z-score matrix of the adjusted BMD-genes expression. In each figure, the first row refers to the BMD-genes signature obtained using Coxnet, the second row using ADMMnet.

By inspecting the heatmaps in Figure S1, we identified two groups of genes (e.g., *PPDZK1, LRP2, PCM1, TMEM26, BCL2, AFF3*) and (e.g., *FUT3, FGFR4, CDC7, RRM2, SPC25, PKMYT1, UBE2C, TROAP*). The first group contains genes such that the lower is their expression the worse is the patient prognosis, the other group contains genes such that the higher is their expression the worse is the patient prognosis. There are however other genes for which the separation of the z-scores in the two risk groups is less evident, as already noticed also in Ahmad and Fröhlich (2017). Figure S2 shows similar behavior and group of genes, reducing the noise in the heatmaps. In this case we identified the same group of genes and few others of interest. Among the latter, for *AURKA* the higher is the expression the worse is the prognosis, as also shown in Jiang et al. (2010).

Second, we show the results obtained using the DAD-screening. In this case, to select $\left\{x_{j},j\in I_{DAD}\right\}$ we used the DAD screening to reduce the dimensionality of the full dataset from *p* to *d*_*DAD*_ < *p*, for different thresholds *d*_*DAD*_ = 100, 200, …, 2, 000. Then, as before, we further reduced the model size down to $d^{\prime }<d_{DAD}$ by fitting a network-based methods for each fixed threshold *d*_*DAD*_. We called DAD-genes the high-risk gene signature (i.e., those genes with regression coefficients ${\hat{\beta }}_{I_{DAD}}\neq 0$). Different choices of the threshold *d*_*DAD*_ = 100, 200, …, 2, 000 lead us to slightly different, but usually overlapping, DAD-gene lists. As before, the significance of the DAD-gene lists were assessed on the testing dataset, *D*. From our analysis we observed that the log-rank test *p*-values were able to separate the high and low risk group of patients with a significance lower than 0.01 only for some range of thresholds. As expected, log-rank *p*-value associated to the DAD-genes are not as strong as the corresponding *p*-values associated to the BMD-genes, suggesting that DAD-screening is not competitive in terms of prediction power with respect to the BMD-screening. Therefore, the information available from the literature should not be neglected and DAD-screening should be used to find potential candidate biomarkers and predict survival only when no other (or very limited) information is available. Anyway, our results also show that the performance of DAD-screening improves when two integrated omic types (mRNA+CNAs) are used instead of the simple gene expression (mRNA) profiles.

Finally, we discuss the results obtained using the BMD+DAD-screening. In this case, to select $\left\{x_{j},j\in I_{BMD+DAD}\right\}$ we merge the two above mentioned-screenings $\left\{x_{j},j\in I_{BMD}\cup I_{DAD}\right\}$ using different thresholds *d*_*DAD*_ = 100, 200, …, 2, 000 when adding the DAD contribution. Such subsets of genes reflect the bio-medical knowledge available from previous studies (BMD part) and also incorporate additional information contained in the data under analysis (DAD part). Analogously to the previous cases, we fitted a network-based Cox regression model in order to further reduce the feature space from *d*_*BMD*+*DAD*_ to *d*′ and to select the high-risk genes or potential biomarkers (i.e., genes with regression coefficients ${\hat{\beta }}_{I_{BMD+DAD}}\neq 0$). We called this signature BMD+DAD-genes. As before, the significance of the BMD+DAD-gene lists was evaluated on the testing dataset, *D*, in terms of *p*-values of the log-rank test for each value of the threshold.

Moreover, in order to understand the BMD and the DAD contribution to the BMD+DAD-genes we subdivided the BMD+DAD-genes in:

- genes-HEFaIMp-high: BMD+DAD-genes that match the genes selected by HEFaIMp tool with *p*-value < 0.05;
- genes-HEFaIMp-low: BMD+DAD-genes that match the genes selected by HEFaIMp tool with *p*-value>0.05;
- genes-no-HEFaIMp: BMD+DAD-genes that are not covered by HEFaIMp tool.

Genes in group (a) are those included in the BMD-screening; genes in group (b) are presented in HEFaIMp but their evidence was not sufficiently strong to let them be included in the BMD-screening. However, our analysis reinforce the evidence that they could be related to breast cancer. By contrast, genes identified in group (c) might be important for the process of novel biomarker discovery since they represent potential biomarkers not previously identified as associated to breast cancer.

Tables S3, S4 show the results obtained from the combination of BMD+DAD-screening and network-penalized methods (Coxnet and ADMMnet) for different thresholds *d*_*DAD*_ = 100, 200, …, 2, 000. From these results, we observed that the log-rank test *p*-value associated to the BMD+DAD-genes on the testing dataset is better (i.e., smaller) than the corresponding *p*-value obtained using the BMD-genes and DAD-genes in both cases investigated (mRNA and mRNA+CNAs data). Therefore, the BMD+DAD-screening outperforms the other two screenings allowing: (i) better separation between high-and-low-risk groups and (ii) identification of novel potential biomarkers. Moreover, our results also confirm that our prediction capability further improves when two omic layers (mRNA + CNAs) are used instead of a single omic layer (mRNA). See also Figure S3 for the combination of BMD+DAD-screening and Coxnet and Figure S4 for the fusion of BMD+DAD-screening and ADMMnet.

**Figure 3:**
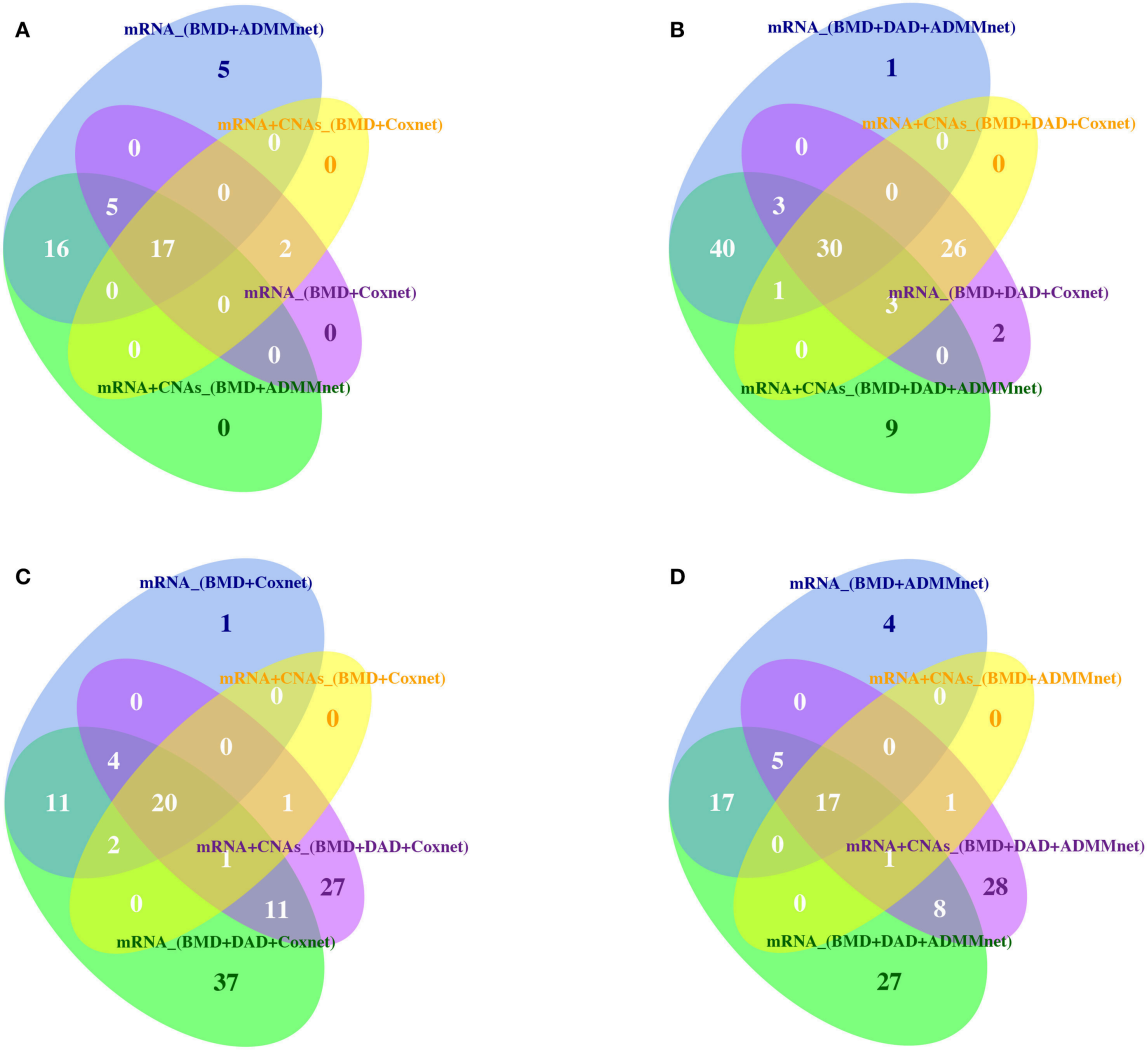
Venn diagrams are used to illustrate **(A)** the intersection of BMD-genes by using Coxnet and ADMMnet with mRNA expression data and the integration of mRNA data and CNAs profiles; **(B)** the intersection of BMD+DAD-genes by using Coxnet and ADMMnet with mRNA expression data and the integration of mRNA data and CNAs profiles; **(C)** the intersection of BMD-genes and BMD+DAD-genes by using Coxnet with mRNA expression data and the integration of mRNA data and CNAs profiles; **(D)** the intersection of BMD-genes and BMD+DAD-genes by using ADMMnet with mRNA expression data and the integration of mRNA data and CNAs profiles.

Then, Tables S5, S6 show the list of BMD+DAD-genes selected from each screening-network approach by using mRNA expression data and the integration of mRNA and CNAs data, respectively. Tables S5, S6 also show the number of times each gene in the signature was selected when changing the threshold and the network methods. We observed that the BMD+DAD-genes create a consensus gene-set signature that is quite robust with respect to the choice of the threshold and can be potentially highly associated with breast cancer prognosis. In particular, *AFF3, ARVCF, AURKA, BCL2, C17orf78, EXPH5, FEZF2, FGFR4, FUT3, LRP2, PDZK1, PKMYT1, REL, SPC25, TMEM26, TROAP, UBE2C* were identified by using both mRNA expression data and mRNA+CNAs data. For these genes the frequency of the occurrence is equal to 20 corresponding to the number of threshold used in our analysis. Finally, to further evaluate the robustness of gene signatures we used Venn diagrams (see Figure 3). From this figure we observed that the overlaps between screening and network methods is quite good, although there are specificities that explain the better performance of one combination with respect to another. Moreover, Figure 3 also show that the BMD+DAD-screening selects novel potential disease risk genes that the simple BMD-screening ignores.

A more comprehensive analysis of these candidate genes is described out in the following section.

### 3.3. Pathway exploration

**Figure 4:**
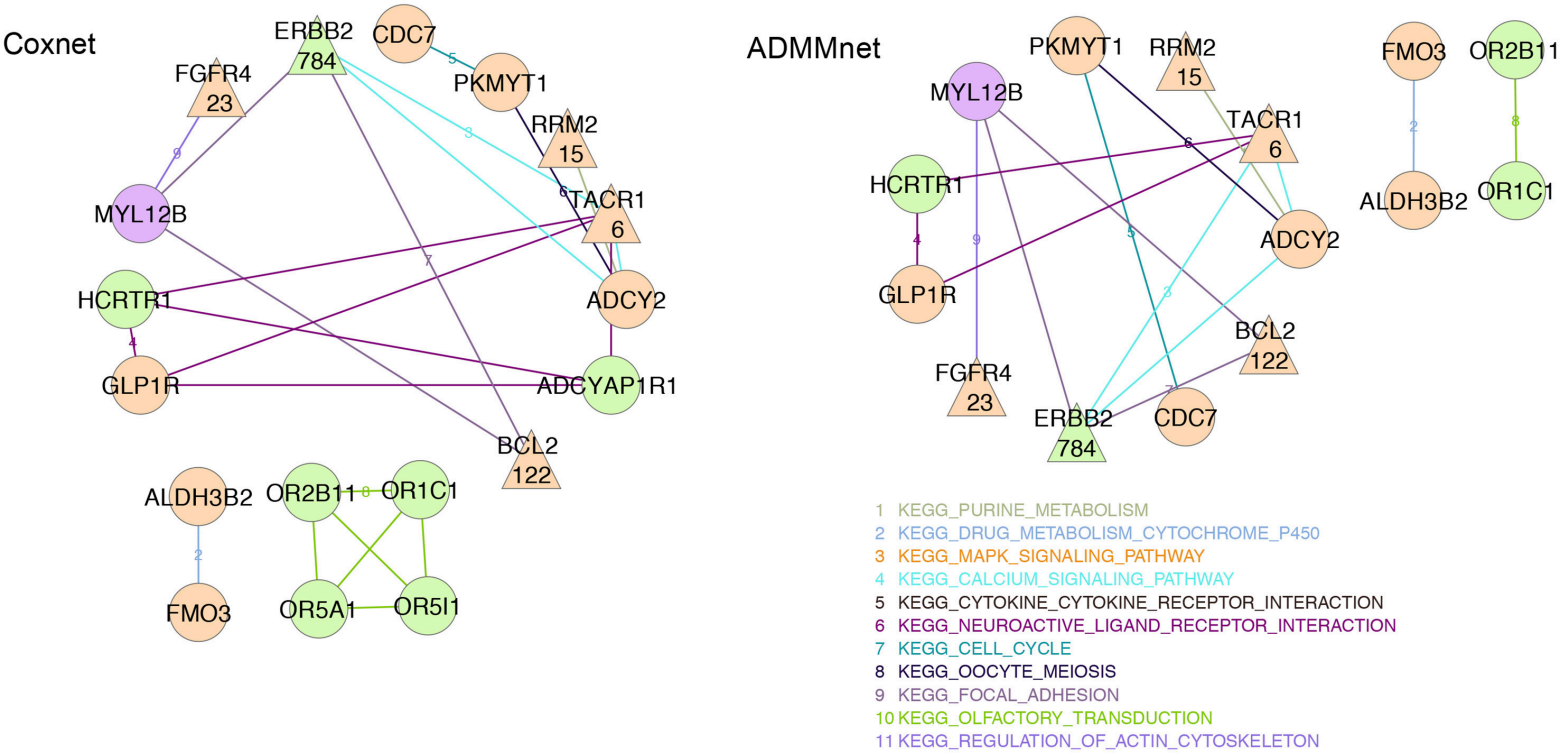
mRNA expression data: BMD+DAD-screening network using Coxnet (left) and ADMMnet (right) methods with mRNA expression data and the integration of mRNA data and CNAs profiles. Non isolated genes are represented as nodes in the network, then a link a drawn between two (adjacent) genes when the two genes belong to the same KEGG pathway. We use different colors for KEGG pathways and three colors to identify the type gene: orange color for genes-HEFaIMp-high, green color for genes-HEFaIMp-low, purple color for genes-no-HEFaIMp. Triangular-shaped nodes indicate the genes identified in literature as breast-cancer associated genes. The number of papers is also reported in the triangular nodes.

**Figure 5:**
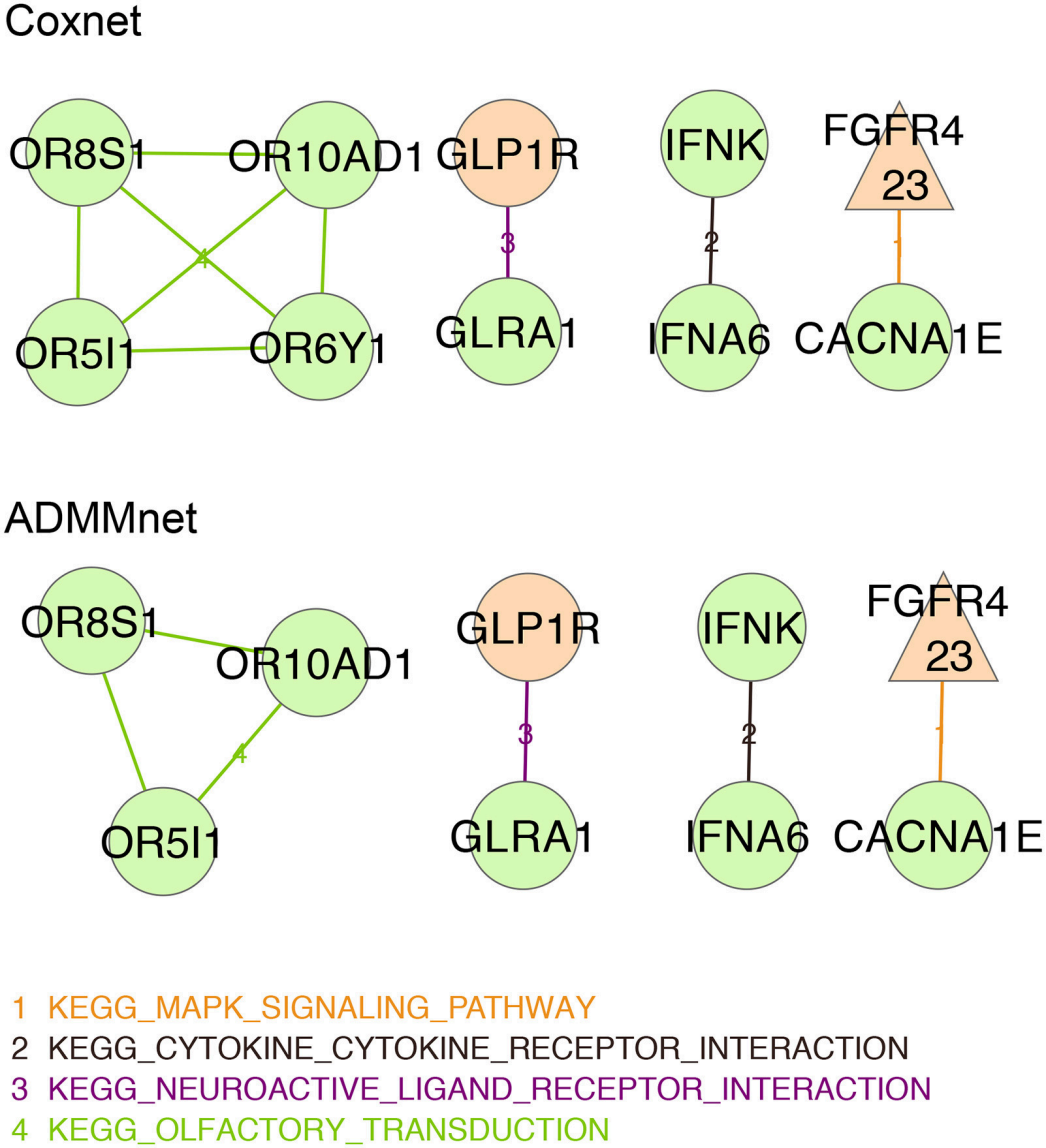
Integration of mRNA data and CNAs profiles: BMD+DAD-screening network using Coxnet (top) and ADMMnet (bottom) methods with mRNA expression data and the integration of mRNA data and CNAs profiles. Non isolated genes are represented as nodes in the network, then a link a drawn between two (adjacent) genes when the two genes belong to the same KEGG pathway. We use different colors for KEGG pathways and three colors to identify the type gene: orange color for genes-HEFaIMp-high, green color for genes-HEFaIMp-low, purple color for genes-no-HEFaIMp. Triangular-shaped nodes indicate the genes identified in literature as breast-cancer associated genes. The number of papers is also reported in the triangular nodes.

In order to better understand and interpret the inferred gene signatures, in this section we report the results of the KEGG pathways analysis performed on the not-isolated genes in the signature (as described in section 2.6). To this purpose we considered the BMD+DAD gene lists identified using Coxnet and ADMMnet models with both mRNA data and the integration of mRNA+CNAs to build the final pathway networks reported in Figures 4, 5. We used such networks to easily visualize the gene-gene interactions and the KEGG pathways involved in such interactions. Each node corresponds to a gene and the edges represent the KEGG pathways shared by the linked genes. Different colors for nodes have been used to indicate genes-HEFaIMp-high (orange), genes-HEFaIMp-low (green) or genes-no-HEFaIMp (purple) as defined in section 3.2. Therefore orange nodes represent the BMD contribution to the signature and green and purple nodes the DAD contribution, not yet captured in the BMD list. Note that some of the genes colored in orange might be also be retrieved from the data under analysis (as DAD-genes), however in this context we want to underline and make sense of the novel information not yet considered.

Figure 4 shows the gene-networks built on the genes identified by Coxnet and ADMMnet respectively using mRNA data. From the color of the nodes, we can infer that most of (but not all) the genes come from the BMD contribution (i.e., orange nodes). Hence, confirming that the BMD+DAD screening allows us to identify few genes that the BMD screening ignores. Moreover, our analysis allows us to further investigate the KEGG pathways the involved genes belong to. In particular, a gene shown in both networks is *BCL2*, which accordingly to Cotterill (1999) has already been mentioned in 122 publications showing its importance in breast cancer. *BCL2* functions to prevent apoptosis and it is a tumor-related gene that has the potential to further improve individualization of patient management, by predicting response to chemotherapy, hormonal therapy and radiotherapy (Joensuu et al., 1994; Hamilton and Piccart, 2000). In addition, as showed in both the networks in Figure 4, *BCL2* is involved in the *KEGG focal adhesion* pathway together with *MYL12B* and *ERBB2*. Extensive studies relate the *KEGG focal adhesion* pathway to breast cancer since it plays critical roles in integrin-mediated signal transduction and also participates in signaling by other cell surface receptors. *KEGG focal adhesion* pathway is also involved in angiogenesis during embryonic development and cancer progression (Parsons, 2003; Cohen and Guan, 2005). In Zhao and Guan (2011), the authors also show the role of this pathway in cells migration and metastatic breast cancer. From the color of the three genes involved in this pathway (in both the networks in Figure 4), it results that even if *MYL12B* is not in the genes-HEFaIMp-high list (the node color is purple), it can play an important role in breast cancer. Indeed, *MYL12B* is involved in the regulation of cell morphology and recent studies have shown the link between such gene and cancer progression (Gurda et al., 2015). Another relevant gene reported in both networks is the fibroblast growth factor receptor-4 (*FGFR4*), which has been widely investigated as one of the major causes of disease progression in estrogen- and progesterone-receptor-positive tumors and in tumors with high lymph-node involvement (Jaakkola et al., 1993), confirming its relationship with breast cancer. Other cancer biomarkers have been reported in both networks with exactly the same pathway edges, which underline their important role in the disease and the accuracy of our algorithm. For example, *CDC7* and *PKMYT1* belong to the *KEGG cell cycle* pathways which is one of the most commonly disrupted pathways in cancer (Chang et al., 2003; Kastan and Bartek, 2004). Similarly *RRM2* and *ADCY2* belong to the *KEGG purine metabolism* pathway whose disruption is often linked with transformation and progression of cancer (Weber, 1983; Pedley and Benkovic, 2017). Overall, our results show that from the pathway analysis of the gene signatures using mRNA data, it is possible to investigate not only the genes involved in the progression of the disease but also the relative pathways which may include novel biomarkers.

Figure 5 shows the networks corresponding to the genes identified by Coxnet and ADMMnet respectively using the integration of mRNA data and CNAs values. The majority of the nodes identified in those networks are green (i.e., with *p*-value > 0.05) which means that by using integrated data both Cox-methods select an higher number of DAD genes than before. It is worthy to note that the two networks are almost identical except for Coxnet that selects one more gene (*OR6YI*) from the *KEGG olfactory transduction* pathway (see Figure 5, Coxnet). This pathway has a functional role in the development and/or progression of melanoma and it may even contribute to tumorigenesis (Ranzani et al., 2017). Both networks report *OR5I1, OR10AD10*, and *OR8S1* as part of the *KEGG olfactory transduction* pathway and they are olfactory receptors that have been linked with the promotion of cancer cell invasiveness and metastasis emergence (Sanz et al., 2014). The *KEGG neuroactive ligand receptor interaction pathway* has been identified in all the four networks (Figure 5). However, a new gene is reported in both the data integration networks that was not reported in the mRNA network, i.e., *GLRA1*. Such gene has been mentioned in several cancer studies as involved in cancer development (Murakami and Hirano, 2008; Kreisler et al., 2010). Two new pathways have been identified by using the integrated data: *KEGG cytokine cytokine receptor interaction* pathway and *KEGG mapk signaling* pathway. Both pathways are essential for cancer-immune evasion in melanoma cells since they regulate a variety of cellular activities including proliferation, differentiation, survival, and death (Lin and Karin, 2007; Kim and Choi, 2010).

In conclusion, we can confirm that by using either mRNA data or the integration of mRNA data and CNAs values, our algorithm is able to identify genes already known to be associated with breast cancer as well as new potential candidate markers and disrupted pathways. As a consequence, either methods can be used for the analysis of cancer pathways depending on the availability of the biological information about the disease under investigation.

### 3.4. Further analysis of potential breast cancer biomarkers

In order to further exploit the relevance of the potential novel biomarkers we have identified with our analysis, we present a gene enrichment integrating somatic mutation using the genes-HEFaIMp-low and no-genes-HEFaIMp lists. Our aim is to better understand the biological relevance and make sense of the genes that were ignored when using the BMD-screening, but were found significant using the BMD+DAD-screening. More precisely, we match the two sublists of BMD+DAD-genes with the Catalog Of Somatic Mutations In Cancer COSMIC (Forbes et al., 2010).

Exploring this additional source of information, some high-risk mutated genes (variant type-missense mutations) in breast cancer are retrieved. Among genes-HEFaIMp-low, an interesting protein coding gene is *EXPH5* (Exophilin 5). This protein has been identified as an important prognostic gene for breast cancer. In particular, this gene is connected to the missense variant (GAG → GTG) which is associated with differential methylation, gene expression, and survival of TCGA breast cancer patients (Shilpi et al., 2017). Another important protein coding gene is *FGFR4* (Fibroblast Growth Factor Receptor 4) which is an essential kinase critical for the proliferation and survival of basal-like breast cancer cells. In particular, this gene mediates cancer cell survival via the activation of PI3K/AKT signaling. Moreover, FGFR4 and FGF19 autocrine signaling may serve as a novel potential therapeutic target for the treatment of refractory basal-like breast cancers (Tiong et al., 2016). *GLP1R* (Glucagon Like Peptide 1 Receptor) is a further protein coding gene expressed in human breast cancer tissue. In particular, the activation of *GLP1R* attenuates breast cancer cells proliferation by inhibiting NF-κB activation and target gene expression (Hirata et al., 2013). Moreover, the protein coding gene *MSX2* (Msh Homeobox 2) is also implicated in breast cancer. It is an important regulator of melanoma cell invasion and survival. Its cytoplasmic expression was identified as prognostic biomarker in malignant melanoma patients (Gremel et al., 2011). Finally, the protein coding gene *TMEM26* (Transmembrane Protein 26) is another important gene expressed in ERα-positive and -negative breast cancer cell lines. In particular, patients who received aromatase inhibitor treatment tend to have a higher risk of recurrence when the expression of *TMEM26* is low. Moreover, *TMEM26* negatively regulates the expression of integrin β1, which is an important factor involved in endocrine resistance (Nass et al., 2016). Among no-genes-HEFaIMp, an important protein coding gene is *ACTL9* (Actin Like 9). An important paralog of this gene is ACTL7A which is implicated in diverse cellular processes, including vesicular transport, spindle orientation, nuclear migration, and chromatin remodeling. In particular, this gene is involved in a risk locus for breast cancer at 9q31.2 (chromosomal position) that provide evidence of an association between variants mapping to 6q25.1 (chromosomal position) and breast cancer risk in subjects of European ancestry (Fletcher et al., 2011). Another important protein coding gene is MYL12B (Myosin Light Chain 12B). Myosin regulatory subunit plays an important role in regulation of both smooth muscle and nonmuscle cell contractile activity via its phosphorylation and it is implicated in cytokinesis, receptor capping, and cell locomotion. In particular, it is predominantly expressed in Triple-Negative Breast Cancer (Ziegler et al., 2014). Among its related pathways there are the *Semaphorin interactions* and *Focal Adhesion* pathways. An important paralog of this gene is MYL12A. The protein coding gene SLC22A25 is also detected by COSMIC. This gene has been identified as hub gene into the mechanisms of gene regulation during breast cancer (Emmert-Streib et al., 2014). An important paralog of this gene is SLC22A9.

## 4. Conclusions

In this work, we combine variable screening procedures and network-penalized Cox models for high-dimensional survival data aimed to reduce the size of initial dataset to a moderate size and to determine pathway structures and potential biomarkers involved in cancer progression. By using these approaches, it is possible to obtain a deeper insight of the gene-regulatory networks and investigate the gene signatures related to the breast cancer survival time in order to understand how patient molecular features can influence survival in cancer. Breast cancer is used as illustrative example, however the proposed methods can be used for different types of cancers.

We illustrate the capabilities of our approaches to predict patient survival using METABRIC dataset. First we used one out of three different screenings methods: biomedical driven screening, data-driven screening and a combination of the two. Then, using the biological network, as prior information network, we performed network-based Cox model to identify specific signatures of genes and the corresponding pathways associated to breast cancer prognosis. Finally we used Kaplan-Meier curve and log-rank test to validate the goodness of the prediction. Hence, while the screening methods recruit the features with the best marginal utility to reduce the dimensionality of the data, the network incorporates the pathway information used as a prior knowledge network into the survival analysis. Overall, we can conclude that (i) the BMD-screening confirms previous results on independent dataset (Iuliano et al., 2016); (ii) the DAD-screening shows good performance in absence of any previous information but it is sub optimal with respect to the BMD-screening; (iii) the BMD+DAD-screening allows to discover novel potential biomarkers for breast cancer that are disregarded by the BMD-screening and improve the BMD-screening in terms of prediction capabilities. Moreover, we also illustrate how to extend the proposed methodologies, initially sought for gene expression data, to the case when two omic data types are available on the same set of patients. In particular, we compared the results obtained by our procedures using only mRNA expression values with those obtained by integrating mRNAs and CNAs. From our results, we can conclude that the use of two omic layers always outperforms the results obtained with a single omic.

Finally, we investigated the potential relevance of the BMD+DAD-genes we have detected. Our results show that they are often connected known cancer genes and are significantly enriched in biological processes and pathways that are involved in breast cancer, or annotated in cancer mutations databases such as COSMIC. Although this computational analysis does not guarantee that such genes can be considered biomarkers, they make sense of biological processes involved in breast cancer progression and provide a strong suggestion toward the need of future studies for their biological validation.

It is clear that the proposed procedures can be applied to different cancer types to obtain a more accurate investigation of the development and progression of the disease. In fact, from one hand breast cancer represents one of the types of cancer for which there is a wide knowledge accumulated in the literature. Nevertheless, the BMD+DAD-screening shows that there is still space for improvements and for novel discoveries. On the other hand, the information available for some types of cancers might not be so accurate. Therefore, methods such as DAD-screening might be useful to provide a good level of analysis.

The results obtained in this work open interesting scenarios for future developments. First, we have shown that the use of two omic layers improves prediction capabilities, therefore the integration of data from multiple omics (e.g., structural variations, methylation or other epigenetic markers and/or metabolomics) into the screening procedure could also provide a more accurate investigation and prevent the limitations of current methods. The possibility of combine together different types of omics or other co-data is expected to further improve the results. Second, in order to support clinicians with a more concrete and biomedical perspective, the proposed procedures should be further extended in order to include also clinical and therapeutical information for each patients. Such information will allow to better stratify the patients in a study and can provide a better characterization of the diseases. Unfortunately, to this regard we note that standard network methods such as Coxnet and ADMMnet do not include procedures for patients stratification in current implementation. This limitation has to be addressed in future works. Third, in order to facilitate the use of the proposed methodology for the analysis of different cancer datasets, it is necessary to implement an interactive user-friendly interface where all preprocessing and normalization steps, as well as the those described in Algorithm 1 can be carried out in terms of a easy point-and-click approach.

## Author contributions

AI and AO prepared the computational codes and carried out all of the statistical analysis. CA, ID, and PL initiated and coordinated the work, guided the study design, supervised all data curation and analysis, and finalized all study conclusion. CA, ID, and PL are equal contributors. All the authors wrote, reviewed and approved the final manuscript.

### Conflict of interest statement

The authors declare that the research was conducted in the absence of any commercial or financial relationships that could be construed as a potential conflict of interest.
