# Supplementary material for: Combining Pathway Identification and Breast Cancer Survival Prediction via Screening-Network Methods
Source: Front Genet. 2018 Jun 14;9:206. doi: 10.3389/fgene.2018.00206 (PMC6011013; doi:10.3389/fgene.2018.00206)
Supplement: Supplementary file 8 [file Image_2.PDF]

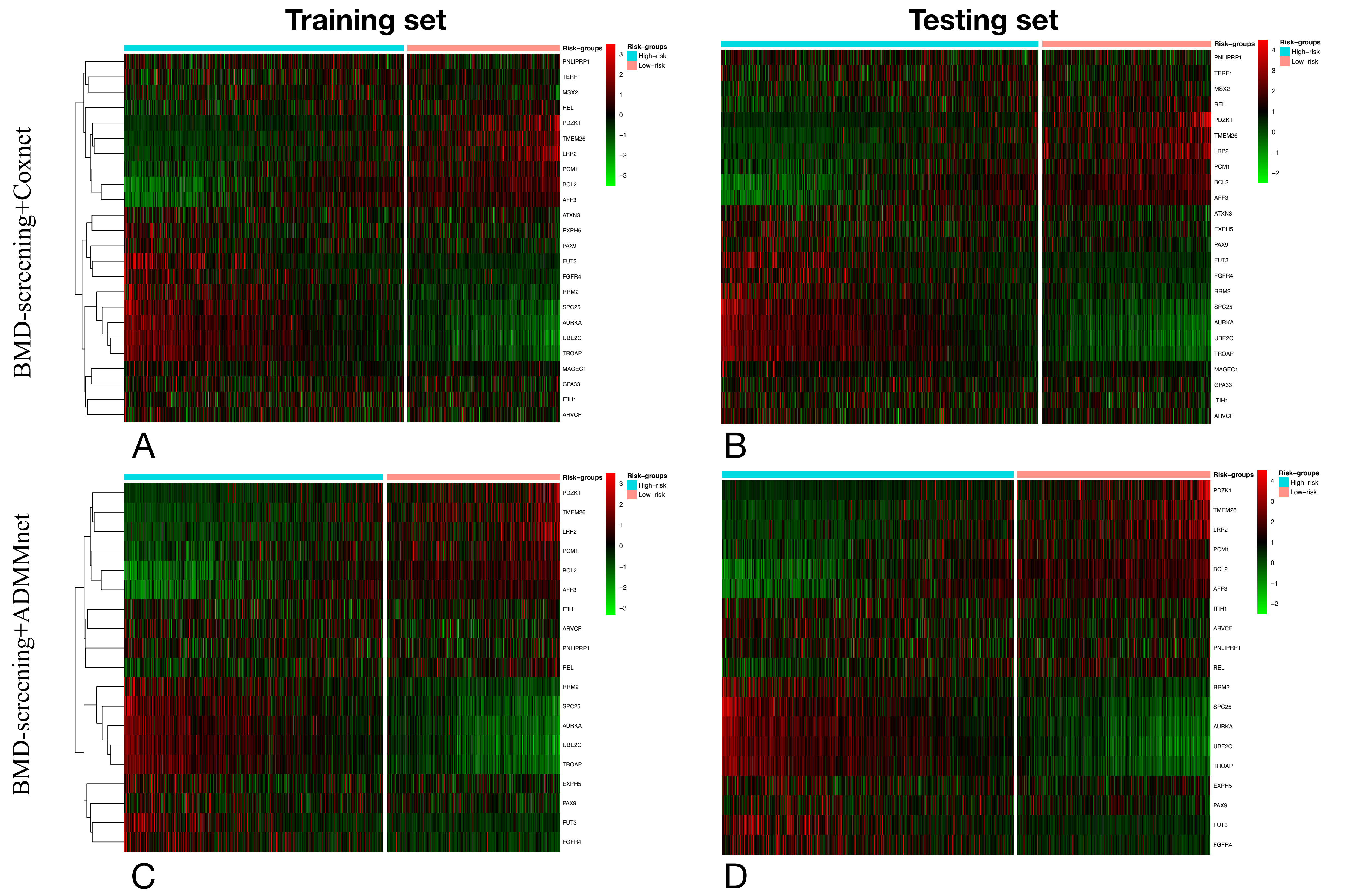

Figure S2. Heat-maps of the BMD-genes obtained applying BMD-screening+Coxnet (A and B) and BMD-screening+ADMMnet (C and D) in the training set (T) and the testing set (D) for the combination of mRNA data and CNA profiles. The genes in the horizontal direction are clustered in the same order in both sets (T and D). Z-scores of the gene expression measurements are used. Z-scores larger than 3.5 were set to 3.5 and Z-scores smaller than -3.5 were set to -3.5. Red color indicates a high level of expression in breast cancer and green color indicates a low level of expression. The patients are divided in high-risk and low-risk groups (i.e., patients with bad and good prognosis).
