# Supplementary material for: Combining Pathway Identification and Breast Cancer Survival Prediction via Screening-Network Methods
Source: Front Genet. 2018 Jun 14;9:206. doi: 10.3389/fgene.2018.00206 (PMC6011013; doi:10.3389/fgene.2018.00206)
Supplement: Supplementary file 10 [file Image_4.PDF]

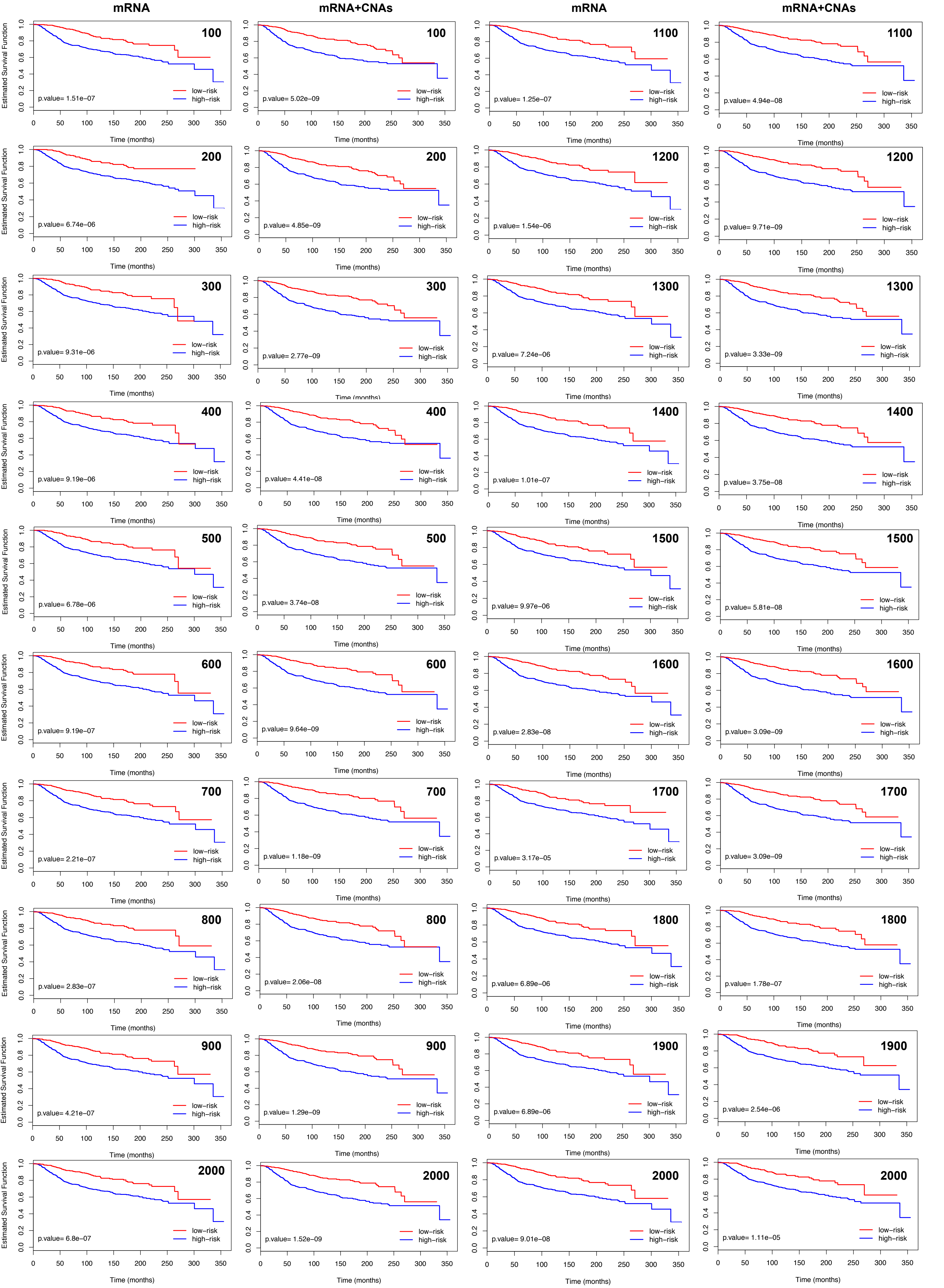
mRNA
mRNA+CNAs
mRNA
mRNA+CNAs
Estimated Survival Function
Time (months)
low−risk
high−risk
100 p.value= 1.51e−07
100 p.value= 5.02e−09
1100 p.value= 1.25e−07
1100 p.value= 4.94e−08
200 p.value= 6.74e−06
200 p.value= 4.85e−09
1200 p.value= 1.54e−06
1200 p.value= 9.71e−09
300 p.value= 9.31e−06
300 p.value= 2.77e−09
1300 p.value= 7.24e−06
1300 p.value= 3.33e−09
400 p.value= 9.19e−06
400 p.value= 4.41e−08
1400 p.value= 1.01e−07
1400 p.value= 3.75e−08
500 p.value= 6.78e−06
500 p.value= 3.74e−08
1500 p.value= 9.97e−06
1500 p.value= 5.81e−08
600 p.value= 9.19e−07
600 p.value= 9.64e−09
1600 p.value= 2.83e−08
1600 p.value= 3.09e−09
700 p.value= 2.21e−07
700 p.value= 1.18e−09
1700 p.value= 3.17e−05
1700 p.value= 3.09e−09
800 p.value= 2.83e−07
800 p.value= 2.06e−08
1800 p.value= 6.89e−06
1800 p.value= 1.78e−07
900 p.value= 4.21e−07
900 p.value= 1.29e−09
1900 p.value= 6.89e−06
1900 p.value= 2.54e−06
2000 p.value= 6.8e−07
2000 p.value= 1.52e−09
2000 p.value= 9.01e−08
2000 p.value= 1.11e−05

Figure S4. Kaplan-Meier plots of mRNA data and the adjusted mRNA data based on CNAs profiles. For each case, patient samples were separated into two groups according to the predicted risk scores from the selected genes by combining BMD+DAD-screening and network Cox regression method (ADMMnet). High-risk group is labelled in blue and low-risk group is labelled in red. The X-axis represents time and the Y-axis represents survival rate.
